# Neuropsychological Performance: How Mental Health Drives Attentional Function in University-Level Football Athletes

**DOI:** 10.3390/sports13030061

**Published:** 2025-02-20

**Authors:** Sacha Assadourian, Dima Daher, Catherine Leclerc, Antony Branco Lopes, Arnaud Saj

**Affiliations:** 1 Department of Psychology, University of Montréal, Montréal, QC H3C 3J7, Canada; sacha.assadourian@umontreal.ca (S.A.); dima.daher@umontreal.ca (D.D.);; 2 Centre Interdisciplinaire de Recherche sur le Cerveau et L’apprentissage (CIRCA), Université de Montréal, Montréal, QC H3C 3J7, Canada; 3 CRIR/Institut Nazareth et Louis-Braille du CISSS de la Montérégie-Centre, Longueuil, QC J4K 5G4, Canada; 4 Spectre Biotech, 75008 Paris, France

**Keywords:** psychological profile, attentional profile, student athlete, QEEG, mental health

## Abstract

This preliminary study investigates the potential relationship between electrophysiological profiles measured by quantitative electroencephalography (QEEG) and attentional performance in 34 university American football players. QEEG data revealed patterns associated with burnout, chronic pain, and insomnia among the athletes. Attentional performance was generally average, but players exhibited faster reaction times in the alertness task without warning, fewer errors in the sustained attention task, and lower scores in the divided attention task, favoring visual information over auditory information. Significant negative correlations emerged between QEEG profiles associated with burnout, ADHD, depression, and anxiety and specific attentional subcomponents. These findings suggest a link between mental health-related brain activity and attentional performance. In a clinical context, they emphasize the need for early detection and intervention in mental health problems. This might improve cognitive performance and well-being in athletes. However, due to the small sample size and the lack of a control group, these results are considered preliminary, and further research is required to confirm and expand on these associations.

## 1. Introduction

Sports performance is not limited to physical strength and training. The mental health of athletes holds equal importance, but it can be weakened by high athletic demands, personal life, and academics [1]. Mental health disorders such as burnout, ADHD, insomnia, chronic pain, depression, and anxiety are common in athletes and may occur more frequently than in the general population [2,3,4,5]. These conditions manifest through fatigue, inattention, sleep disturbances, pain, and emotional distress [6]. Traditional screening methods, such as questionnaires, have limitations, as athletes may be reluctant to report mental health issues due to stigma [7,8]. Quantitative electroencephalography (QEEG) provides an objective method of detecting mental health conditions [9,10]. It relies on EEG, a non-invasive measure of brain activity in real time using electrodes placed on the scalp. QEEG quantifies brainwave frequencies, enabling comparisons with normative data and identifying patterns associated with conditions such as burnout [11], ADHD [12], insomnia [13], pain [14], depression [15], and anxiety [16] and providing deeper insights into brain activity. While QEEG patterns can reflect certain neurophysiological activity observed in individuals with mental health conditions, these patterns themselves are not diagnostic and should be interpreted solely as potential indicators of underlying neurophysiological states rather than clinical conditions.

Cognition, which includes perception, attention, memory, and decision-making, is essential in interacting with the environment and plays a critical role in sports performance [17]. Elite athletes often display superior cognitive abilities compared to non-athletes, highlighting its importance in sporting success [17]. American football, with its strategic demands and rapid decision-making, highlights diverse cognitive skills required across different sports. This study focuses primarily on attention, a critical cognitive component in athletes. Players utilize three attentional functions: (1) alertness, to quickly react to unexpected changes in the game; (2) divided attention, to monitor multiple elements simultaneously; and (3) sustained attention, to maintain attention throughout the match. Attentional functions can be evaluated using standardized assessment tools, which help provide insights into cognitive abilities.

Mental health conditions are closely linked to cognitive performance, which is crucial in sports success [4,18]. Conditions like burnout, ADHD, insomnia, chronic pain, depression, and anxiety can impair key cognitive functions such as attention, memory, and executive functioning. For example, burnout reduces executive function, memory, attention, and processing speed [19]. ADHD affects reaction time variability and working memory [20]. Insomnia compromises attention and working memory [21]. Chronic pain affects inhibition and memory [22]. Depressive disorders impact attention, memory, and executive functions, even during recovery [23]. Finally, anxiety disorders impair perception, attention, memory, and executive functions [24]. These impairments can prevent athletes from achieving optimal performance outcomes. Studies suggest that QEEG can identify symptoms related to anxiety and inattention [25]. However, further investigation is warranted to clarify the link between mental health and attentional performance in elite athletes. This study investigates the link between electrophysiological profiles and attentional performance in athletes. We hypothesize that players with QEEG profiles resembling those associated with burnout, ADHD, insomnia, chronic pain, depression, or anxiety will exhibit lower attentional performance. While this study’s objectives address an important gap in understanding athlete mental health and cognition, we acknowledge that methodological constraints may limit the broader applicability of our findings. Nevertheless, these preliminary results provide valuable insights to guide future research directions.

## 2. Materials and Methods

Thirty-four university American football players from the U Sports league aged 19 to 25 (M = 22.06 years, SD = 1.43) participated in this study. The distribution by sports year was as follows: 18% in the first year, 21% in the second, 53% in the third, 6% in the fourth, and 3% in the fifth. Participants trained an average of 5.38 sessions per week (SD = 1.21). The measurements were conducted during the off-season period, in the absence of scheduled field training sessions, allowing for controlled testing conditions without interference from acute physical fatigue or training effects.

Inclusion and exclusion criteria were applied. All participants had to be active university team members and at least 18 years old. Participants were excluded if they had known neurological or psychiatric conditions, including burnout, ADHD, insomnia, chronic pain, depression, or anxiety, or if they were taking medications that could affect attentional performance or EEG results. Uncorrected sensory deficits (auditory or visual) and excessive substance use (alcohol or drugs) were also exclusionary criteria. Hair density was considered exclusionary when it prevented accurate EEG measurement due to poor electrode impedance. All participants signed written informed consent forms prior to participation. The study received approval from the Ethics Board for Research in Education and Psychology of the University of Montreal (Project No. CEREP-21-037-P) and complied with the Declaration of Helsinki regarding research involving human subjects.

QEEG profiles were measured using the advanced Spectre Biotech^®^ EEG system. This system includes a 16-channel headset with dry, flexible ThinkPulseTM polymer electrodes and reference electrodes typically placed on the mastoids. The setup allows for quick installation (<2 min) and the reliable acquisition of brain activity. The wireless Bluetooth Low Energy (BLE 4.2) connectivity ensures participant mobility during recording. Data points were excluded based on established thresholds for signal-to-noise ratio, movement artifacts, and electrode impedance to ensure optimal recording quality. Detailed information about specific quality thresholds and exclusion criteria are available upon request. From the 204 total patterns recorded (6 patterns × 34 players), 151 patterns (74%) met the quality criteria for analysis, while 53 patterns (26%) were excluded due to data points failing to meet established quality criteria. Data analysis was conducted using a computer equipped with Spectre Biotech^®^ software (Version 1.0.0), which performed real-time spectral power analysis using a fast Fourier transform with Hanning windowing. Participants underwent a standardized 3 min resting-state EEG recording with both eyes open and closed. The QEEG profiles were analyzed based on established patterns linked to mental health conditions. These included Alpha peak frequency for burnout [11]; right frontal Gamma power for ADHD [12]; frontoparietal Beta3 power for insomnia [13]; prefrontal Beta and Gamma1 power for chronic pain [14]; phase coherence between the frontal and temporoparietal regions for chronic depression [15,26]; and prefrontal alpha asymmetry for anxiety [16]. Each profile was expressed as percentiles and z-scores, classified as follows: within the norm (0–24; z-score, 0 to +1 SD); slightly elevated (25–49; z-score, +1 to +1.66 SD); moderately elevated (51–74; z-score, +1.67 to +2 SD); or severely elevated (75–100; z-score, +2 SD and above).

Attentional performance was assessed using three tasks from the TAP (Test of Attentional Performance) [27]. Alertness assesses the ability to increase attention in anticipation of an event. Participants have to react as quickly as possible to the appearance of a simple visual stimulus by pressing a key, sometimes preceded by a warning tone. Performance is measured by the reaction time and the reaction time stability; the accuracy of the answers is secondary (given the ease of the task). The task, divided into four blocks, examines intrinsic alertness (without a warning tone) and phasic arousal (with a warning tone). Divided attention (form 1): assesses the ability to process multiple sources of information simultaneously. Participants have to react simultaneously to specific visual patterns and auditory sequences. Performance is measured by the number of omissions; the reaction time is secondary. The task involves 100 visual and 200 auditory stimuli. Sustained attention (color or form): assesses the ability to maintain attention over an extended period. Participants have to identify visual stimuli based on common characteristics (color or form) with the previous stimulus. Performance is also measured by the number of omissions; the reaction time is secondary. This 15 min task involves 450 stimuli and 54 targets. Scores are expressed as percentiles and classified as follows [28]: extremely low (<2); below average (2 to 8); low average (9 to 24); average (25 to 74); high average (75 to 90); above average (91 to 97); or extremely high (98 to 100). Most information regarding the validity and reliability of these tasks can be found in the TAP manual. For example, the construct validity of the alertness task is driven by the reaction speed in simple conditions (correlation: 0.82–0.92). The divided attention task is influenced by multiple factors, such as the visual task speed (correlation: 0.41), visual exploration speed (negative correlation: −0.66, reflective of a trade-off between speed and accuracy), and auditory task speed and accuracy (correlations: 0.68–0.81). The split-half reliability is excellent for reaction times in alertness (>0.90) and errors in divided attention (0.92). The test–retest stability is good for the alertness task (correlation: 0.81) and acceptable for the divided attention task (correlations: 0.44–0.64). The average interval between pre- and post-test results was 25.06 days (SD = 5.75 days; range = 18 to 43).

Before correlation analysis, the data normality was assessed using the Shapiro–Wilk test for each variable. Since the QEEG profiles and many attention scores did not follow a normal distribution, descriptive statistics were adapted accordingly. QEEG profiles were presented as frequencies per category, while the central tendency for all attentional task results was described by the average for normally distributed scores and the median for non-normally distributed scores. As QEEG scores were not normally distributed, nonparametric Spearman correlation tests were used to examine the relationship between QEEG profiles and attentional performance. Given the hypothesized inverse relationship, a one-tailed test was used. Given the exploratory nature of this study, we did not apply corrections for multiple comparisons to avoid being overly strict in identifying potential relationships that could guide future confirmatory research. All analyses were conducted using Jamovi software (Version 2.6.23).

## 3. Results

### 3.1. QEEG Profiles

QEEG patterns reveal neurophysiological profiles that have been associated in the previous literature with experiences of mental health such as burnout, chronic pain, and insomnia (see Table 1). While these patterns share similarities with those observed in clinical populations, they should be interpreted as potential neurophysiological indicators rather than diagnostic markers. These data range from showing slightly to severely elevated neurophysiological patterns. It is important to note that these patterns, while sharing similarities with those observed in clinical populations, should not be interpreted as diagnostic indicators. Missing values resulted from unusable EEG data caused by environmental interference, including room conditions, surrounding devices, and sweating after training.

### 3.2. Attentional Performance

Overall, the attentional performance was within the average range (see Table 2). Participants demonstrated faster reaction times in the alertness task without an auditory warning, slower reaction times to auditory information in the divided attention task, and fewer errors in the sustained attention task.

### 3.3. Correlations Between QEEG Profiles and Attentional Performance

Exploratory analyses revealed correlations between neurophysiological activity patterns and specific attentional subcomponents (see Table 3). QEEG profiles resembling those observed in burnout were as follows: slower reaction times to visual information in the divided attention task (rho = −0.431, *p* < 0.05), more omissions (rho = −0.347, *p* < 0.05), and greater instability in reaction times in the sustained attention task (rho = −0.415, *p* < 0.05). QEEG profiles resembling those observed in ADHD were as follows: slower reaction times in the alertness task without (rho = −0.450, *p* < 0.05) and with auditory warnings (rho = −0.469, *p* < 0.05) and more errors in the divided attention task (rho = −0.454, *p* < 0.05). QEEG profiles resembling those observed in depression were as follows: more omissions of auditory information in the divided attention task (rho = −0.423, *p* < 0.05). QEEG profiles resembling those observed in anxiety were as follows: more instability in reaction times in the alertness task without auditory warnings (rho = −0.35, *p* < 0.05) and more omissions in the divided attention task (rho = −0.40, *p* < 0.05). No significant correlations were found between QEEG profiles resembling those observed in chronic pain or insomnia and attentional performance.

## 4. Discussion

This study examined QEEG profiles and attentional performance in university American football players, revealing three key findings: (1) a high prevalence of neurophysiological patterns linked to mental health conditions such as burnout, ADHD, insomnia, and chronic pain; (2) overall average attentional performance with notable strengths (e.g., faster reaction times in alertness tasks) and weaknesses (e.g., slower reaction times in auditory divided attention tasks); and (3) significant correlations between QEEG profiles and attentional subcomponents, highlighting the potential cognitive impacts of specific neurophysiological patterns.

### 4.1. QEEG Profiles and Mental Health Prevalence

The presence of these patterns suggests potential risk factors or underlying neurophysiological states that warrant further clinical investigation through formal psychiatric assessment.

QEEG profiles resembling those observed in burnout were found in 42.2% of players, with 26.9% showing severe neurophysiological patterns, consistent with Sorkkila et al. [5], who reported a 40.3–45.3% prevalence range. QEEG profiles resembling those observed in ADHD appeared in 45% of participants, with 17% showing moderate patterns, aligning with Cage et al. [29], who observed a 16% diagnostic rate and 46.2% prevalence based on self-reports, highlighting the need for timely management given the heightened injury risks for athletes with ADHD [30]. QEEG profiles resembling those observed in insomnia were found in 50% of participants, with 36% showing severe patterns, consistent with Mah et al. [31], who reported a prevalence of 39.1–51%, emphasizing the importance of addressing sleep hygiene, given its link to suicidal ideation [32]. QEEG profiles resembling those observed in chronic pain appeared in 36% of participants, with 32% showing severe patterns, consistent with Christopher et al. [3], who noted a prevalence of 34.9%, reinforcing the need for preventive measures like preseason pain assessments to mitigate risks [33]. QEEG profiles resembling those observed in depression were identified in 14.3% of participants, with 10.7% showing severe patterns, aligning with Wolanin et al. [34], who reported a prevalence of 15.6–21%. No QEEG profiles resembling those observed in anxiety were observed, diverging from Gulliver et al. [35], who reported a 14.7% prevalence, possibly reflecting methodological or population differences.

Discrepancies in prevalence rates compared to other studies may result from differences in methodology (e.g., subjective vs. objective assessments), environmental factors, and variations in how “prevalence” is defined (e.g., risk vs. diagnosis). Despite exclusion criteria for diagnosed conditions, QEEG revealed neurophysiological patterns similar to those observed in clinical populations, suggesting potential subclinical states or risk factors that warrant monitoring, even in apparently healthy athletes.

### 4.2. Attentional Performance in Relation to Athletic Expertise

The attentional performance among participants was within the average range, with faster reaction times in the alertness task without a warning tone, suggesting heightened readiness to respond to sudden visual stimuli, consistent with studies showing superior reaction times in elite athletes [36,37,38], though some report conflicting findings [39,40]. This may reflect perceptual expertise enabling athletes to focus on critical information [41,42], while factors like field position and visual behaviors may also contribute [41,43]. Fewer errors in the sustained attention task indicated slightly better focus over time, aligning with some evidence of enhanced sustained attention in athletes [44], although this is debated [39]. The slower reaction times in the auditory component of the divided attention task highlight a potential area for improvement, as divided attention is crucial in processing verbal instructions while monitoring the environment. However, the research on divided attention in elite athletes remains scarce. 

### 4.3. Correlations Between QEEG Profiles and Attentional Performance

The correlations between QEEG patterns and attentional subcomponents suggest that neurophysiological activity resembling that observed in mental health conditions can influence cognitive performance. QEEG profiles resembling those observed in burnout showed slower reaction times during multitasking and increased errors in sustained attention, consistent with findings that burnout impairs speed and accuracy in attention-demanding tasks [19]. QEEG profiles resembling those observed in ADHD correlated with slower reaction times in alertness tasks, regardless of auditory warnings, and higher multitasking error rates, supporting evidence of the impact of ADHD on reaction time consistency and accuracy [20]. QEEG profiles resembling those observed in depression were linked to more multitasking errors, aligning with research on the negative effects of depression on attention and task engagement [23]. QEEG profiles resembling those observed in anxiety were linked to unstable reaction times in alertness without warnings and more errors in multitasking. Research shows that anxiety leads to a narrowing of attentional focus and difficulty processing multiple stimuli [24], with behavioral inhibition further slowing reactions in some athletes [45]. No significant correlations emerged for QEEG profiles resembling those observed in insomnia nor chronic pain, suggesting subtler or no cognitive effects, which warrants further investigation. While multiple correlations were examined between QEEG markers and attentional subcomponents, we opted not to apply corrections for multiple comparisons, given the exploratory nature of this study. This approach allowed us to identify potential relationships that warrant further investigation in confirmatory research, though the results should be interpreted with appropriate caution.

### 4.4. Implications and Applications

QEEG provides objective insights into brain activity, complementing self-reports by detecting subtle neurophysiological patterns resembling those observed in mental health conditions like burnout, ADHD, insomnia, and chronic pain. These patterns, while not diagnostic, serve as sensitive screening tools to identify at-risk athletes. Integrating QEEG into questionnaires and comprehensive evaluations facilitates early detection and targeted interventions. Regular QEEG monitoring, combined with efforts to ensure data quality by addressing factors like environmental interference and electrode impedance, can support personalized athlete care. Tailored approaches might include cognitive training, adjusted rest schedules, and EEG neurofeedback (EEG-NFB), which has shown efficacy in enhancing attention [46], sleep [47], pain management [48], burnout [49] and depression [50].

Neurophysiological patterns can guide personalized interventions through EEG neurofeedback training. This approach could help athletes learn to self-regulate their brain activity patterns towards optimal attentional states. Protocols could be customized based on individual QEEG profiles, with regular monitoring allowing for the dynamic adjustment of training parameters to maximize effectiveness.

The findings on attentional performance revealed strengths in visual alertness and weaknesses in divided attention, underscoring the need for training programs to enhance multitasking skills critical in high-pressure scenarios. Regular QEEG and neuropsychological brief assessments could track attentional trends, refining interventions and optimizing performance.

The correlations between QEEG profiles and attentional performance further suggest that neurophysiological patterns may predict cognitive challenges, aiding the design of holistic, evidence-based strategies by sports psychologists and neuropsychologists to improve attentional capacity and resilience. An integrated approach is recommended, as follows: (1) Prevention—normalizing mental health discussions to reduce stigma; (2) Screening—using QEEG with questionnaires to identify at-risk athletes; (3) Evaluation—conducting thorough clinical assessments for diagnosis; (4) Intervention—developing tailored cognitive and mental health strategies; and (5) Monitoring—regular QEEG assessments to refine care strategies. 

### 4.5. Limitations and Future Directions

The small sample size (*N* = 34) and single-site recruitment significantly constrain generalizability and reduce statistical power. Future studies should incorporate larger, more diverse samples across multiple sites and competitive levels.

The absence of control groups limits our ability to determine whether QEEG attention patterns are specific to football players or characteristic of athletes in general. Future research should include both non-athlete controls and athletes from other sports to differentiate sport-specific effects from general athletic training effects.

Multiple factors impacted data quality, leading to 26% data loss from electromagnetic noise, electrode conductivity issues, and physiological factors. Future studies should implement more stringent environmental controls, including electromagnetic shielding, standardized post-activity rest periods, and rigorous electrode impedance monitoring.

The laboratory-based TAP battery lacked ecological validity, potentially underestimating real-world attentional demands during competition. Kalen et al. [51] emphasize that tasks mirroring real-world sports demands better capture sport-specific attentional expertise, which may not transfer to standardized clinical assessments. Future studies should develop sport-specific cognitive assessments that better reflect competitive play’s attention requirements.

The absence of formal mental health assessments limited QEEG pattern correlations. Future research should incorporate gold-standard clinical questionnaires (e.g., the Beck Depression Inventory and the Insomnia Severity Index).

Neurophysiological patterns may manifest differently across various recording systems due to differences in technical aspects. The exclusive use of one QEEG system may limit the results’ generalizability. Future studies should conduct cross-validation using multiple QEEG systems to establish generalizability across different technological platforms.

Off-season testing may not capture cognitive demands during competition. Future research should implement longitudinal assessments throughout the athletic season to examine how attentional performance fluctuates under varying competitive pressures.

Longitudinal designs would provide help in tracking how neurophysiological patterns change over the course of an athletic season and career and determining the directionality of the observed correlations, i.e., whether changes in neurophysiological activity precede changes in well-being or vice versa.

These methodological improvements could strengthen our understanding of relationships between mental health, neurophysiological activity, and athletic performance, ultimately enhancing mental health support in sports programs.

## 5. Conclusions

This study achieved its primary objective of investigating the relationships between QEEG patterns and attentional performance in athletes, providing preliminary evidence linking mental health-related neurophysiological patterns to cognitive function. While QEEG and attention tasks offer valuable insights into the relationship between brain function and cognitive abilities, the findings should be interpreted with caution. Further research involving larger samples, control groups, clinical assessments, and a broader cognitive battery is essential in confirming and expanding upon these results. Combining objective measures like QEEG with subjective assessments could facilitate the early identification of mental health concerns, though refining these methods through continued research is necessary. Personalized cognitive training programs based on an athlete’s QEEG and attentional patterns may help improve performance, but these interventions require careful tailoring with expert involvement.

## Figures and Tables

**Table 1:** QEEG profiles showing characteristics commonly found in mental health conditions.

Conditions	Within the Norm	Slightly Elevated	Moderately Elevated	Severely Elevated
Burnout	57.7%	11.5%	3.8%	26.9%
ADHD	56%	28%	17%	
Insomnia	50%	7%	7%	36%
Chronic pain	64%	4%		32%
Depression	85.7%	3.6%		10.7%
Anxiety	100%			

Note: percentages represent the proportion of players falling into each category based on their QEEG profiles.

**Table 2:** Results for attention tasks.

Task	Measure	Score Percentiles	Labels
Alertness			
No warning tone	Reaction time	Md = 81.59%	High average
	Standard deviation	Md = 63.67%	Average
Warning tone	Reaction time	M = 66.19%	Average
	Standard deviation	M = 60.96%	Average
Divided attention			
Auditory	Omissions	Md = 50%	Average
	Reaction time	Md = 3.66%	Below average
	Standard deviation	M = 38.21%	Average
Visual	Omissions	MD = 38.21%	Average
	Reaction time	M = 41.39%	Average
	Standard deviation	M = 42.91%	Average
Total	Errors	Md = 34.46%	Average
	Omissions	Md = 46.04%	Average
Sustained attention	Errors	Md = 81.59%	High average
	Omissions	Md = 46.04%	Average
	Reaction time	M = 43.93%	Average
	Standard deviation	M = 42.45%	Average

**Table 3:** Spearman correlations between QEEG profiles and attentional performance.

Table	Measure	Burnout	ADHD	Insomnia	Chronic Pain	Depression	Anxiety
Alertness							
No warning tone	Reaction time	0.140	−0.450 *	0.068	0.185	−0.129	−0.184
	Standard deviation	−0.224	−0.298	0.107	0.067	0.085	−0.340 *
Warning tone	Reaction time	−0.321	−0.469 *	−0.032	0.099	0.055	−0.199
	Standard deviation	0.010	−0.381	−0.204	−0.065	0.208	−0.185
Divided attention							
Auditory	Omissions	0.188	−0.264	−0.267	−0.097	−0.423 *	−0.382 *
	Reaction time	−0.011	−0.256	0.032	0.111	−0.005	−0.257
	Standard deviation	−0.233	−0.330	0.020	−0.087	0.282	−0.255
Visual	Omissions	−0.282	−0.274	−0.026	0.003	0.018	−0.344 *
	Reaction time	−0.431 *	−0.354	0.060	0.044	−0.064	−0.085
	Standard deviation	−0.227	0.048	0.083	0.140	0.019	0.156
Total	Errors	−0.304	−0.454 *	0.285	0.045	0.018	0.289
	Omissions	−0.122	−0.233	−0.115	−0.097	−0.164	−0.345 *
Sustainedattention	Errors	−0.308	0.042	0.280	−0.046	0.145	0.200
	Omissions	−0.347 *	0.034	0.046	−0.019	−0.038	−0.284
	Reaction time	−0.194	0.120	0.008	0.021	0.066	−0.185
	Standard deviation	−0.415 *	0.281	0.155	0.131	−0.019	0.085

Note: Hₐ is a negative correlation. * *p* < 0.05; unilateral.

## Data Availability

The data in this study are not publicly available, as they are still undergoing additional analyses. Once these analyses are complete, the data will be made available upon reasonable request to the corresponding author.
